# Rare *TP53* variant associated with Li-Fraumeni syndrome exhibits variable penetrance in a Saudi family

**DOI:** 10.1038/s41525-018-0074-3

**Published:** 2018-12-19

**Authors:** Musa AlHarbi, Nahla Mubarak, Latifa AlMubarak, Rasha Aljelaify, Mariam AlSaeed, Amal Almutairi, Weal AlJabarat, Fatimah Alqubaishi, Lamia Al-Subaie, Nada AlTassan, Cynthia L. Neben, Alicia Y. Zhou, Malak Abedalthagafi

**Affiliations:** 1 Comprehensive Cancer Center, King Fahad Medical City, Riyadh, 11525 Saudi Arabia; 2 Genomics Research Department, Saudi Human Genome Project, King Fahad Medical City and King Abdulaziz City for Science and Technology, Riyadh, 11525 Saudi Arabia; 3 Division of Genetics, Department of Pediatrics, King Abdulaziz Medical City, Riyadh, 11525 Saudi Arabia; 4 Department of Genetics, King Faisal Specialist Hospital and Research Center, Riyadh, 11525 Saudi Arabia; 5 Color Genomics, Burlingame, CA 94010 USA; 6 Department of Pathology, Brigham and Women’s Hospital, Harvard Medical School, Boston, MA 02115 USA

## Abstract

Li-Fraumeni syndrome (LFS) is an inherited, autosomal-dominant condition that predisposes individuals to a wide-spectrum of tumors at an early age. Approximately 70% of families with classic LFS have pathogenic variants in the tumor suppressor gene *TP53* that disrupt protein function or stability. While more than 70% of pathogenic variants in *TP53* are missense variants, the vast majority occur very infrequently, and thus their clinical significance is uncertain or conflicting. Here, we report an extremely rare *TP53* missense variant, c.799C > T (p.Arg267Trp), identified in a 2-year-old Saudi proband diagnosed with choroid plexus carcinoma (CPC) and six of his first- and second-degree relatives. CPC is frequently found in families with LFS, and this is the first detailed report of a family with this variant. Intriguingly, the proband’s father is homozygous for *TP53* c.799C > T and phenotypically normal at 39 years of age. While loss of *TP53* heterozygosity is often observed in tumors from individuals with LFS, homozygous germline *TP53* pathogenic variants are rare. Based on our analysis of this single family, we hypothesize that *TP53* c.799C > T has low or variable penetrance for LFS, with predisposition to the development of CPC. The observations from this family have furthered our understanding of the phenotypic variability that may be caused by one variant of *TP53*, even in the same family, and suggest that other factors (genetic and/or environmental) may play a role in mechanism of disease manifestation in LFS.

## Introduction

Li-Fraumeni syndrome (LFS [MIM: 151623]) is an inherited, autosomal-dominant condition that predisposes individuals to a wide-spectrum of tumors presenting in childhood, adolescence, and adulthood. Nearly half of affected individuals have a cancer diagnosis before age 30 years, and those who survive have an increased risk for multiple primary cancers.^1^ These include early onset breast cancer, soft tissue and bone sarcomas, brain tumors such as choroid plexus carcinoma (CPC), and adrenocortical carcinoma (ACC), each of which carry age and sex-specific risks. The revised Chompret criteria was proposed to identify families with LFS beyond a strict clinical diagnosis by (1) increasing the minimum age of tumor onset and (2) identifying a unique subset of cancers (specifically CPC and ACC) for which genetic testing should be considered, regardless of family history.^2,3^ The only genetic variations definitively associated with LFS are pathogenic variants in *TP53* (ref. ^1^), a tumor suppressor gene whose protein product mediates DNA damage response, apoptosis, and cell cycle arrest. More than 70% of *TP53* pathogenic variants in LFS families are missense variants, most frequently at one of six “hotspot” residues in the DNA binding domain.^4,5^ These commonly occurring missense variants have been associated with early tumor onset and a more highly penetrant phenotype due to adverse gain-of-function effects.^6–8^ However, 69% of missense variants are found less than 10 times in the UMD TP53 database (http://p53.fr),^4^ and thus their clinical significance in uncertain or conflicting.

Here, we add to the heterogeneity of LFS by reporting a rare *TP53* missense variant, c.799C > T (p.Arg267Trp), in seven individuals from one non-consanguineous family from Saudi Arabia. This family meets the revised Chompret criteria for a clinical diagnosis of LFS^2,3^ with a history of brain tumors including CPC, colorectal cancer, and liver cancer. Intriguingly, the proband’s father is homozygous for the respective c.799C > T allele and phenotypically normal at age 39 years. While *TP53* loss of heterozygosity is frequently observed in tumors from individuals with LFS, homozygous germline *TP53* pathogenic variants are rare.

## Results

### Case report

**Fig. 1:**
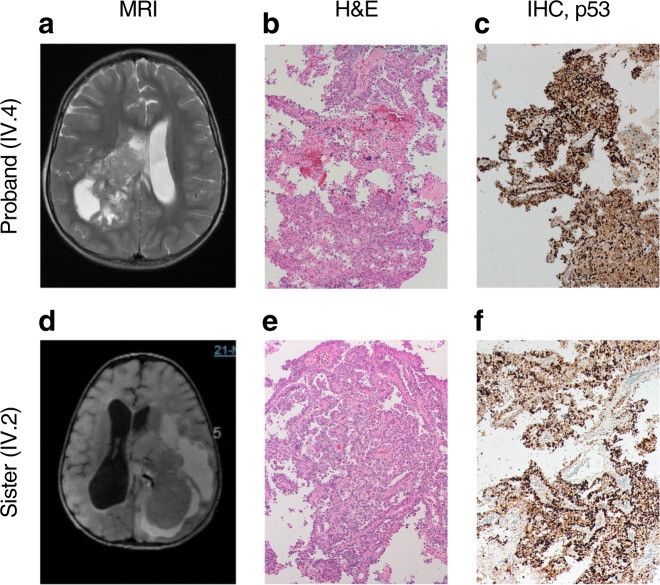
Clinical features and genotypic-phenotypic variability in LFS family. **a** MRI of the proband (IV.4) showed typical choroid plexus carcinoma (CPC) with a large, heterogeneously-enhancing right lateral intra-ventricular mass with foci of calcification and hemorrhaging. **b** Histological section of the proband’s tumor stained with hematoxylin and eosin (H&E) showed increased cellularity with a predominantly solid pattern, nuclear atypia, and increased mitotic activity (magnification 100×). **c** Immunohistochemistry (IHC) staining for p53 of the proband’s tumor showed positive nuclear accumulation (magnification 100×). **d** MRI of the proband’s sister (IV.2) showed CPC. **e** Histological section of the proband’s tumor stained with H&E showed increased cellularity with a predominantly papillary pattern, nuclear atypia, and increased mitotic activity (magnification 100×). **f** Immunohistochemistry staining for p53 of the proband’s sister’s tumor showed positive nuclear accumulation (magnification 100×)

The proband (IV.4) is one of five siblings from a non-consanguineous marriage of parents from different geographical areas. He was initially referred at age 2 years and 7 months with headaches and repeated vomiting for one month. His magnetic resonance imaging (MRI) showed a large, heterogeneously-enhancing right lateral intra-ventricular mass with foci of calcification and hemorrhaging (Fig. 1a), most likely representing CPC. We confirmed differential diagnosis of CPC by histopathology which showed increased cellularity with a predominantly solid pattern, nuclear atypia, and increased mitotic activity (Fig. 1b). Because CPC is frequently found in families with LFS,^9^ our initial pathology review included immunohistochemistry staining for p53. We found strong positive nuclear accumulation in the proband’s tumor sample (Fig. 1c), suggestive of p53 dysfunction and consistent with published reports of LFS tumors.^9,10^ Sanger sequencing of the tumor showed loss of TP53 heterozygosity, which is often observed in tumors from individuals with LFS (Supplementary Figure 1). The proband completed two cycles of ifosfamide, carboplatin, and etoposide (ICE) chemotherapy to reduce tumor size and vascularity before undergoing a right parietal occipital craniotomy and subtotal resection (STR). To date, he has completed six cycles of ICE chemotherapy and is clinically stable, though still under follow-up. His last MRI scans showed residual tumor along the posterior aspect of the right temporal lobe.

The proband’s oldest sister (IV.2) had a similar but more severe phenotype. At age 14 months, she had a STR of a differentially diagnosed CPC (Fig. 1d,e) and received three cycles of ICE chemotherapy. Immunohistochemistry staining for p53 in the tumor showed strong positive nuclear accumulation (Fig. 1f), and sanger sequencing of the tumor showed loss of TP53 heterozygosity (Supplementary Figure 1). Four months later, her MRI scans later showed recurrent CPC with a cystic formation in the left hemisphere. Four years later, her MRI scans showed rapid disease progression with significant increase in tumor size and leptomeningeal metastases to the brain parenchyma. At this point, the family refused any further treatment but agreed to continue with palliative and supportive care. Her neurological condition continued to deteriorate until death at age 7 years. Her cause of death was CPC (WHO grade III) with cerebrospinal fluid cytology positive for malignant cells.

**Fig. 2:**
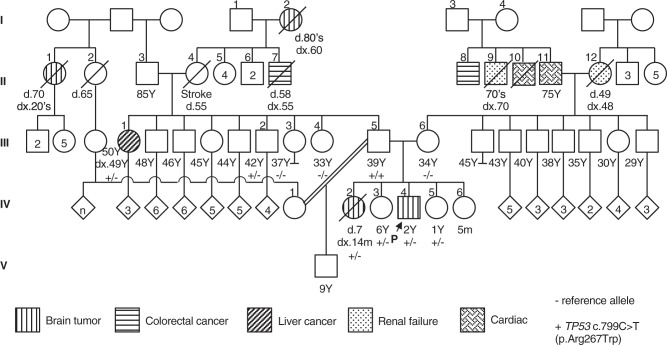
Pedigree indicating the known genotype and phenotype of family members from five generations. “−” and “+” indicate the *TP53* reference and pathogenic (c.799C > T; p.Arg267Trp) allele, respectively. Different symbols indicate the presence of brain tumors, colorectal cancer, liver cancer, renal failure, and cardiac events, as shown in the key

We reviewed the proband’s family history and identified several first- and second- degree relatives affected by tumors reported in association with LFS^11^ (Fig. 2). In addition to the proband’s sister (IV.2), his paternal great-grandmother (I.2) and paternal great aunt (II.1) had brain tumors in adulthood; family history and imaging records suggest these brain tumors were CPCs, however, histopathology reviews were not performed for differential diagnosis. The proband’s paternal aunt (III.1) had liver cancer diagnosed at age 49 years, and his paternal grand-uncle (II.7) had colorectal cancer diagnosed at age 55 years. On his maternal side, the proband’s maternal grand-uncle (II.8) was diagnosed with colorectal cancer at age 70 years.

### Genetic analysis

**Fig. 3:**
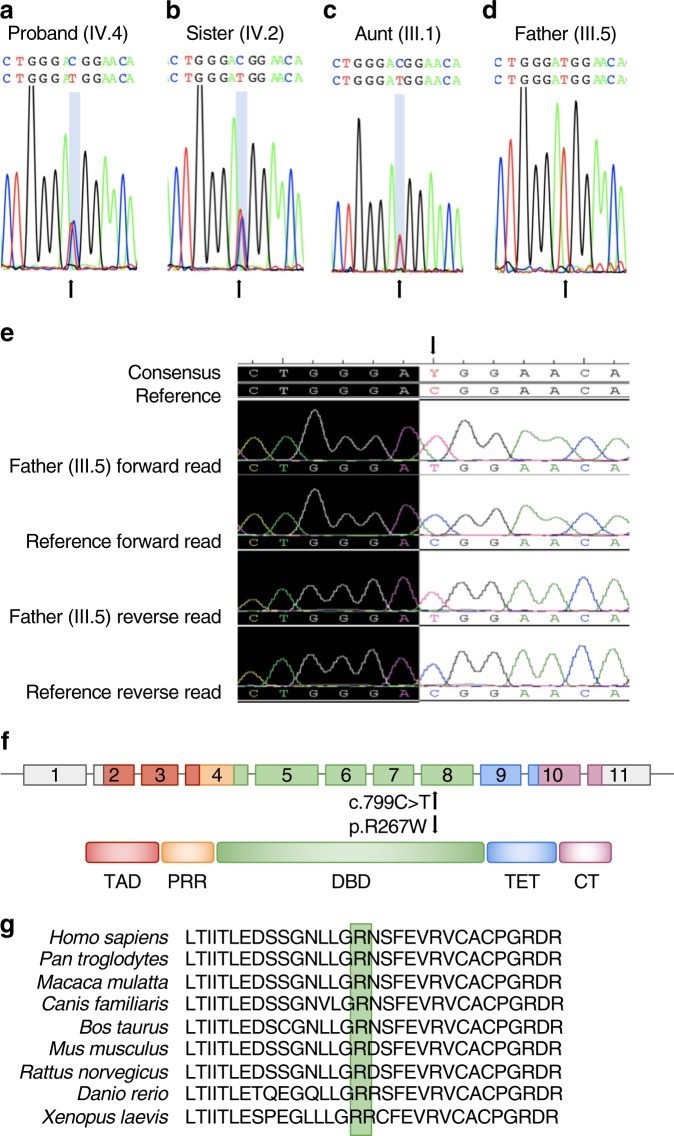
*TP53* pathogenic variant analysis in the LFS family. **a** Chromatograms of DNA extracted from saliva showing the heterozygous c.799C > T pathogenic variant (arrow) in the proband (IV.4), **b** his sister (IV.2), and (**c**) his aunt (III.1) as well as **d** the homozygous c.799C > T pathogenic variant in the proband’s father (III.5). **e** Chromatograms of DNA extracted from peripheral blood showing the homozygous c.799C > T pathogenic variant (arrow) in the proband’s father (III.5) compared to the human genome reference sequence. **f** Schematic diagram of the human *TP53* gene and protein structure. c.799C > T (p.Arg267Trp) is denoted by an arrow. TAD, transactivation domain; PRR, proline-rich region; DBD, DNA-binding domain; TET, tetramerization domain; CT, C-terminal regulatory domain. **g** p.Arg267Trp is conserved in humans, chimpanzee, Rhesus monkey, dog, cow, mouse, rat, zebrafish, and frog

A personal history of CPC, regardless of family history, meets the revised Chompret criteria for LFS, and we recommended genetic testing for the proband and at-risk family members. We identified a heterozygous missense variant c.799C > T in *TP53* in the proband (IV.4; allele fraction, AF 50.24% at 205×) and three of his sisters (IV.2, AF 52.07% at 265×; IV.3, AF 45.23% at 294×; and IV.5, AF 51.48% at 270×) using a 30-gene NGS panel and confirmed by Sanger sequencing (Figs. 3a,b). Two of these sisters (IV.3 and IV.5) are phenotypically normal (Fig. 2); the youngest sister (age 5 months) did not receive genetic testing and is phenotypically normal (Fig. 2). LFS follows an autosomal-dominant inheritance pattern, and as such, we expected either the proband’s father or mother to test positive. While his mother (III.6) does not carry any *TP53* pathogenic variant (Fig. 3c), his father (III.5) is a homozygous carrier of *TP53* c.799C > T (AF 100% at 297×) (Figs. 3d,e). Even more surprising, the father is phenotypically normal with no personal history of cancer at age 39 years. These data suggest that the proband inherited the variant from his father, and we therefore performed genetic testing of second-degree paternal relatives. Of the proband’s three paternal aunts (III.1, III.3, and III.4) who received genetic testing, two paternal aunts (III.3 and III.4) are homozygous for the reference allele and phenotypically normal; the third paternal aunt (III.1) was heterozygous for *TP53* c.799C > T and had liver cancer diagnosed at age 49 years (Fig. 2). The only paternal uncle who received genetic testing (III.2) was also heterozygous for the familial allele (AF 48.84% at 432×) but phenotypically normal (Fig. 2). Although we were unable to perform genetic testing on the proband’s paternal grandparents, these data suggest that at least one of them was a carrier.

We did not identify any other candidate variants (pathogenic, likely pathogenic, or variant of uncertain significance) from the 30-gene panel used for genetic testing of the proband, his siblings, his parents, or his aunts. However, his paternal uncle (III.2) was heterozygous for *BRCA2* c.7534C > T (p.Leu2512Phe). *BRCA2* c.7534C > T (p.Leu2512Phe) has conflicting interpretations of pathogenicity in ClinVar from benign to variant of uncertain significance.^12^ Taken together, this data suggest that *TP53* c.799C > T is the causative allele in this LFS family.

## Discussion

*TP53* c.799C > T maps to a highly conserved region of exon 8 that encodes the DNA binding domain of p53 (amino acids 102-292) (Figs. 3f,g), by which p53 targets genes that control cell cycle arrest (such as *CDKN1A, GADD45, PLK3*, and *MDM2*) and apoptosis (such as *BAX*). This missense variant is predicted to be damaging by SIFT but only possibly damaging by Polyphen2, and ClinVar has conflicting interpretations of pathogenicity, from uncertain significance to likely pathogenic.^13^ Classification of this variant as likely pathogenic is supported by well-established in vitro functional studies. In yeast, human p53 p.Arg267Trp retained at least partial transactivation activity of p53-responsive elements including *BAX, GADD45*, and *MDM2* but had a loss of p21 binding.^6,14^ In human glioblastoma cells, p53 p.Arg267Trp reduced activation of *BAX* and *CDKN1A* reporters by 22 and 23%, respectively, compared to wild type p53 (ref. ^15^). Similarly, p53 p.Arg267Trp did not induce transcription of *CDKN1A* and *PLK3* reporters, among others, or cell death in response to external stimuli in human *TP53* null colorectal carcinoma cells.^16^ Taken together, these data suggest that c.799C > T results in partial loss-of-function.

*TP53* c.799C > T is not listed in the Exome Aggregation Consortium browser or the Genome Aggregation Database but has been reported in the literature as a germline variant in three independent studies. These include an early-onset female breast cancer patient^17^ and a woman age 52 years with multiple tumors (breast fibroadenoma, subependymoma, melanoma *in situ*, and sessile serrated adenoma)^18^ who were heterozygous for c.799C > T. A third individual age 43 years was a compound heterozygote for c.799C > T and c.665C > T (p.Pro222Leu) and had no personal history of cancer.^19^ The Saudi family in this report had no history of breast cancer or melanoma, but similar to the third study,^19^ had three individuals (III.2, IV.3, and IV.5) heterozygous for c.799C > T with no personal history of cancer. Of the three affected individuals in this study, the proband and his oldest sister (IV.1) had CPC diagnosed at a very young age while the proband’s paternal aunt (III.1) had liver cancer at age 49 years. This spectrum of phenotypes and variable penetrance could be due to the remaining transactivation activity of the *TP53* protein product. Alternatively, recent work has identified genetic events that modify the LFS phenotype including intragenic variants, variants of genes in the p53 regulatory pathway, telomere attrition, and copy number variation.^9^ In the targeted 30-gene panel used for genetic testing, we did not identify any large structural variants such as copy number variation in any of the family members who received NGS testing. Our future studies will include analyses of whole genome sequencing of the proband’s nuclear family to identify other candidate variants.

Heterozygous germline pathogenic variants are reported in approximately 70% of classic LFS families,^20^ and homozygosity for germline *TP53* pathogenic variants is extremely rare. To our knowledge, there have only been five previous reports of homozygous germline *TP53* pathogenic variants. Four of the reports were of children in south and southeast Brazil who had the *TP53* c.1010G > A (p.Arg337His) Brazilian founder allele; three children had ACC,^21–23^ and one child had CPC.^23^ The fifth report is of homozygous truncating pathogenic variants in a child age 2 years with CPC and rhabdomyosarcoma.^24^ In stark contrast, in this study, the proband’s father is homozygous for c.799C > T and presents phenotypically normal at 39 years with no personal history of cancer. By age 50 years, men with LFS have a 68% risk of developing cancer, with an average age of onset at 40 years.^25,26^ However, those estimates are based on studies of men with heterozygous variants. Given the spontaneous tumors and premature death observed in *P53* knockout mouse models,^27^ we would expect that the presence of two pathogenic *TP53* alleles in humans would lead to a compound phenotype with increased risk for early onset cancer. It is possible that the proband’s father is germline heterozygous with a somatic variant in his peripheral blood lymphocytes, which could be attributed to classic mosaicism or clonal hematopoiesis associated with aging.^28,29^ However, we did not observe any evidence of retention of the reference allele by NGS (AF at 100%, 297×) or Sanger sequencing (Figs. 3d, e). Unfortunately, we have been unable to obtain additional tissue samples to further test this hypothesis.

Based on the analysis of this single family, we hypothesize that *TP53* c.799C > T has low or variable penetrance for LFS, with predisposition to the development of CPC. CPC accounts for 1 to 4% of all pediatric brain tumors but is frequently found in families with LFS.^9^ Recent reports estimate that between 36 to 63% of individuals with CPC carry a *TP53* pathogenic variant.^9,30–32^ Based on family history and imaging records, we suspect that the proband’s great-grandmother’s (presumed carrier) and paternal grand-aunt’s brain tumors were also CPCs. CPC is extremely rare in adults, however, there have been several recently reported diagnoses in individuals ranging from ages 21 to 73 years.^33–35^ We will continue to follow-up with the proband’s two unaffected carrier sisters and his paternal aunt (III.3) for development of CPC.

CPC has a high incidence of recurrence and metastasis along the central nervous system, and children with TP53-immunopositive CPC, as is the case with the proband and his oldest sister, have a five-year survival rate of 0% compared to those with TP53-immunonegative CPC at 82% (ref. ^9^). With no established curative therapy, intensive surveillance protocols can significantly improve long-term survival rates in children and adults. These protocols use a combination of physical exams, blood tests, and imaging for early tumor detection and reduction of cancer, and a recent panel of LFS experts recommended that surveillance should be offered to all individuals carrying a pathogenic *TP53* variant and individuals who fit the classic definition of LFS, regardless of *TP53* status.^36^ As our understanding of TP53 function and phenotype-genotype correlation in LFS continues to expand, targeted molecular therapies hold the greatest promise for preventing tumor recurrence and prolonging survival. For example, a recent case report demonstrated successful treatment of a 4-month-old female who presented with a recurrent and metastatic CPC through molecular-guided therapy. After 36 months of treatment, her MRI showed 92% tumor reduction, and the metastatic tumor was cleared; she continued to thrive one year after completion of the study.^37^ Targeted therapy options such as this will continue to expand through genetic modeling of LFS and utilization of recently developed methodologies.^38^

In summary, this is the first detailed report of a family with the extremely rare *TP53* missense variant c.799C > T. The younger age of onset and increased disease severity with successive generations suggests the possibility of genetic anticipation, which has been reported in LFS families with *TP53* pathogenic variants.^39–41^ Further analysis are needed to understand how this may influence the mechanism of disease in this family. However, the observations from this family have furthered our understanding of the phenotypic variability that may be caused by one variant of *TP53*, even in the same family, and suggest that other factors (genetic and/or environmental) may play a role in mechanism of disease manifestation in LFS.

## Methods

Written informed consent was obtained for genetic testing of the proband and several members of his family under a research protocol approved by the Institutional Review Board of King Fahad Medical City (Riyadh, Saudi Arabia; #16-300). All family members who provided a saliva sample received a 30-gene next generation sequencing (NGS) panel for detection of pathogenic variants associated with elevated risk of hereditary cancer. NGS panel testing was performed at the Color laboratory (Burlingame, CA) under CLIA (Clinical Laboratory Improvements Amendments, #05D2081492) and CAP (College of American Pathologists, #8975161) compliance.

Sequence reads were aligned against human genome reference GRCh37.p12 with the Burrows-Wheeler Aligner.^42^ Single nucleotide variants (SNVs) and small insertions and deletions (indels, 2–50 bp) were called by the HaplotypeCaller module of GATK3.4. Large structural variants (SVs, >50 bp) were detected using dedicated algorithms based on read depth, paired reads, and split reads. The coverage requirements for reporting were ≥20 unique reads (20×) for each base of the reportable range and ≥50X for 99% of the reportable range. Median coverage typically ranged between 200–300×.

The saliva samples from two family members (paternal aunts III.1 and III.4) repeatedly failed NGS and were instead analyzed by Sanger sequencing. Sanger sequencing confirmation of *TP53* in affected family members was performed through the Color laboratory and at the King Fahad Medical City and King Faisal Specialist Hospital and Research Center. Additional details can be found in the Supplementary File on *npj Genomic Medicine*’s website.

Hematoxylin and eosin staining and immunohistochemistry staining for p53 protein (monoclonal mouse anti-human, Dako, clone DO-7) were performed according to the manufacturer’s instructions by King Fahad Medical City Pathology under CAP accreditation.

## Supplementary information

Supplementary

## Data Availability

The data that support the findings in this study are available on request from the corresponding author (MA). The data are not publicly available as they contain information that could compromise research participant privacy or consent.

## **Supplementary information**

**Supplementary information** accompanies the paper on the *npj Genomic Medicine* website (10.1038/s41525-018-0074-3).
