# Supplementary material for: Rare TP53 variant associated with Li-Fraumeni syndrome exhibits variable penetrance in a Saudi family
Source: NPJ Genom Med. 2018 Dec 19;3:35. doi: 10.1038/s41525-018-0074-3 (PMC6300601; doi:10.1038/s41525-018-0074-3)

SUPPLEMENTARY FIGURE LEGENDS

Supplementary Figure 1. TP53 status of the proband's tumor and the sister's tumor. (**a**) Chromatograms of DNA extracted from the proband's tumor (IV.4) and sister's tumor (IV.2) showing the homozygous c.799C>T pathogenic variant (arrow).

METHODS

Gene Panel Content

The Color Hereditary Cancer Test was used to analyze 30 genes in which pathogenic variants have been associated with an elevated risk for hereditary breast, ovarian, uterine/endometrial, colorectal, melanoma, pancreatic, prostate, and stomach cancer. These genes are *APC, ATM, BAP1, BARD1, BMPR1A, BRCA1, BRCA2, BRIP1, CDH1, CDK4, CDKN2A* (p14ARF and p16INK4a), *CHEK2, EPCAM, GREM1, MITF, MLH1, MSH2, MSH6, MUTYH, NBN, PALB2, PMS2, POLD1, POLE, PTEN, RAD51C, RAD51D, SMAD4, STK11,* and *TP53*. Analysis, variant calling, and reporting focused on the complete coding sequence and adjacent intronic sequence of the primary transcript(s), unless otherwise indicated: in *PMS2*, exons 12-15 were not analyzed. In several genes, only specific positions known to impact cancer risk were analyzed (genomic coordinates in GRCh37): *CDK4* - only chr12:g.58145429-58145431 (codon 24) (ref. 1-3), *MITF* - only chr3:g.70014091 (including c.952G>A),[4–6] *POLD1* - only chr19:g.50909713 (including c.1433G>A),[7,8] *POLE*: only chr12:g.133250250 (including c.1270C>G),[7,8] *EPCAM* - only large deletions and duplications including the 3' end of the gene,[9,10] and *GREM1* - only duplications in the upstream regulatory region.[11–13]

Laboratory Procedures

Laboratory procedures were performed at the Color laboratory under CLIA (Clinical Laboratory Improvements Amendments, #05D2081492) and CAP (College of American Pathologists, #8975161) compliance. DNA was extracted from blood or saliva samples and purified using the Perkin Elmer Chemagic DNA Extraction Kit (Perkin Elmer, Waltham, MA) automated on the Hamilton STAR (Hamilton, Reno, NV) and the Chemagic Liquid Handler (Perkin Elmer, Waltham, MA). The quality and quantity of the extracted DNA were assessed by UV spectroscopy (BioTek, Winooski, VT). High molecular weight genomic DNA was enzymatically fragmented and prepared using the Kapa HyperPlus Library Preparation Kit (Kapa Biosciences, Cape Town, South Africa) automated on the Hamilton Star liquid handler. Target enrichment was performed with an automated (Hamilton Star) hybrid capture procedure using SureSelect XT (Agilent, Santa Clara, CA) probes before being loaded onto the NextSeq 500/550 instrument (Illumina, San Diego, CA) for 150 bp paired-end sequencing.

Variant Interpretation

Variants were classified according to the American College of Medical Genetics and Genomics 2015 guidelines for sequence variant interpretation,[14] and all variant classifications were signed out by a board certified medical geneticist or pathologist. Results were reported as positive if one or more pathogenic or likely pathogenic variant was detected and negative if no variant/and only benign, likely benign, or variant of uncertain significance was detected.

Statistical Analysis

No statistical analysis was necessary for this report.

REFERENCES

1. Wölfel, T. *et al.* A p16INK4a-insensitive CDK4 mutant targeted by cytolytic T lymphocytes in a human melanoma. *Science* **269,** 1281–1284 (1995).
2. Zuo, L. *et al.* Germline mutations in the p16INK4a binding domain of CDK4 in familial melanoma. *Nat. Genet.* **12,** 97–99 (1996).
3. Soufir, N. *et al.* Prevalence of p16 and CDK4 germline mutations in 48 melanoma-prone families in France. The French Familial Melanoma Study Group. *Hum. Mol. Genet.* **7,** 209–216 (1998).
4. Bertolotto, C. *et al.* A SUMOylation-defective MITF germline mutation predisposes to melanoma and renal carcinoma. *Nature* **480,** 94–98 (2011).
5. Yokoyama, S. *et al.* A novel recurrent mutation in MITF predisposes to familial and sporadic melanoma. *Nature* **480,** 99–103 (2011).
6. Ghiorzo, P. *et al.* Prevalence of the E318K MITF germline mutation in Italian melanoma patients: associations with histological subtypes and family cancer history. *Pigment Cell Melanoma Res.* **26,** 259–262 (2013).
7. Palles, C. *et al.* Germline mutations affecting the proofreading domains of POLE and POLD1 predispose to colorectal adenomas and carcinomas. *Nat. Genet.* **45,** 136 (2012).
8. Valle, L. *et al.* New insights into POLE and POLD1 germline mutations in familial colorectal cancer and polyposis. *Hum. Mol. Genet.* **23,** 3506–3512 (2014).
9. Ligtenberg, M. J. L. *et al.* Heritable somatic methylation and inactivation of MSH2 in families with Lynch syndrome due to deletion of the 3' exons of TACSTD1. *Nat. Genet.* **41,** 112–117 (2009).
10. Kovacs, M. E., Papp, J., Szentirmay, Z., Otto, S. & Olah, E. Deletions removing the last

exon of TACSTD1 constitute a distinct class of mutations predisposing to Lynch syndrome. *Hum. Mutat.* **30,** 197–203 (2009).

11. Jaeger, E. *et al.* Hereditary mixed polyposis syndrome is caused by a 40-kb upstream duplication that leads to increased and ectopic expression of the BMP antagonist GREM1. *Nat. Genet.* **44,** 699–703 (2012).
12. Rohlin, A. *et al.* GREM1 and POLE variants in hereditary colorectal cancer syndromes. *Genes Chromosomes Cancer* **55,** 95–106 (2016).
13. Davis, H. *et al.* Aberrant epithelial GREM1 expression initiates colonic tumorigenesis from cells outside the stem cell niche. *Nat. Med.* **21,** 62–70 (2015).
14. Richards, S. *et al.* Standards and guidelines for the interpretation of sequence variants: a joint consensus recommendation of the American College of Medical Genetics and Genomics and the Association for Molecular Pathology. *Genet. Med.* **17,** 405–424 (2015).

Supplementary Figure 1

a

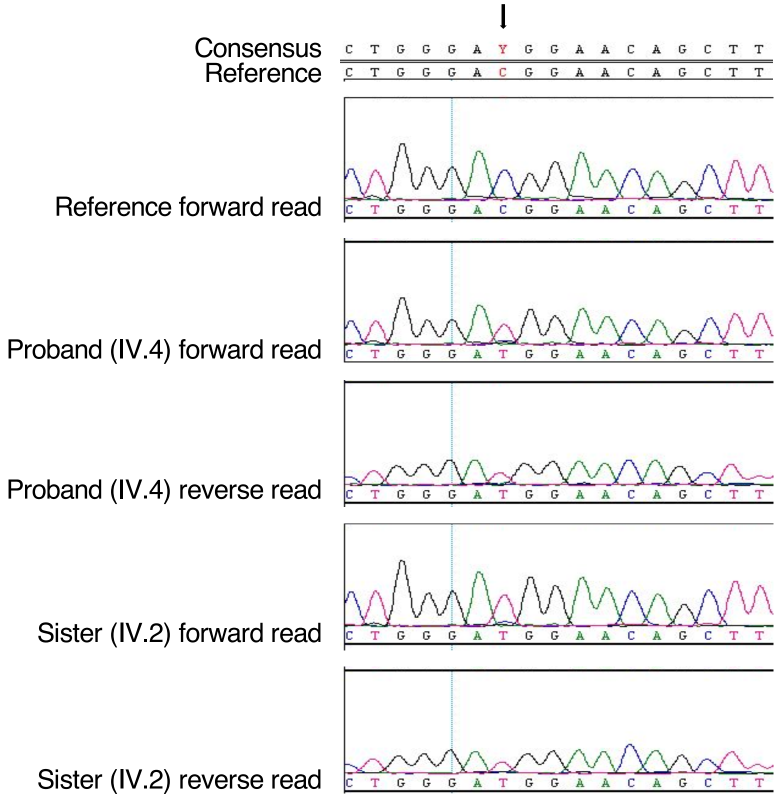

Supplement: Supplementary file 1 — Supplementary [file 41525_2018_74_MOESM1_ESM.pdf]
